# Standardized training assessment of residents in obstetrics and gynecology on entrustable professional activities

**DOI:** 10.1186/s12909-025-06973-7

**Published:** 2025-05-28

**Authors:** Wenjing Zhang, Yiping Hao, Teng Zhang, Xinlin Jiao, Zhonghao Mao, Yuankai Zhang, Dongxiu Zhao, Baoxia Cui

**Affiliations:** 1 Department of Obstetrics and Gynecology, Qilu Hospital of Shandong University, Jinan, 250012 China; 2 Department of Orthopedics, Qilu Hospital of Shandong University, Jinan, 250012 China; 3 Medical Training Office, Qilu Hospital of Shandong University, Jinan, 250012 China

**Keywords:** Residency education, Feedback, EPA, Training assessment

## Abstract

**Background:**

Entrustable professional activity (EPA) presents trainees’ tasks or responsibilities which are entrusted to the unsupervised execution once they have attained sufficient specific competence. EPAs evaluation emphasize the integrity of medical behavior, directly reflecting the clinical ability of trainees and reflect the trust of trainers. Compared with other evalution method, EPAs evaluation is more close to clinical practice and can better reflect clinical competence. Our study conducted a multi-center study in Qilu Hospital of Shandong University to evaluate EPAs among residents. This study was to evaluate EPAs of residents and identify problems in the residency training in Shandong Province, so as to put forward constructive suggestions in futrue.

**Methods:**

This is a cross-sectional study. An assessment survey comprised of 15 EPAs was invented and carried out in Department of Obstetrics and Gynecology, Qilu hospital of Shandong Province. Each resident conducted self-assessment and director-assessment through by internet questionnaire of Wenjuanxing.

**Results:**

In our study, 132 residents and 45 directors were totally enrolled. Both self- and director- assessment had an increased scores trend year by year in each EPAs. However, for PGY1 and PGY2 residents, self-assessment and director-assessment results were relatively consistent. Compared with director-assessment, significant difference was found in self-assessment on admit a patient, select and interpret auxiliary examinations, compose medical documents, perform basic operation and perform health education. Among the four different position of residents (social training residents, entrusted training residents, professional master and permanent staff), permanent staff has the highest EPA scores in this EPAs-evaluation study. In this study, we found significant difference between self-assessment and director-assessment in PGY2 and PGY3 (*p* < 0.05).

**Conclusions:**

EPAs are practically useful in assessment of OB &GYN residents. EPA4, EPA7, EPA1 and EPA15 need to be improved in future training. Individualized feedbacks should be provided based on the difference between self-assessment and director- assessment.

**Supplementary Information:**

The online version contains supplementary material available at 10.1186/s12909-025-06973-7.

## Introduction

In China, standardized training for resident doctors is an important process to be qualified doctors [1, 2]. Due to the particularity of obstetrics and gynecology diseases, obstetrics and gynecology residential training should pay a lot of attention to the cultivation of the comprehensive ability of residents. Quantification the clinical ability requirements according to the growth rules of residents, and formation of a training and assessment mode with strong operability and disciplinary characteristics are extremely important to obstetrics and gynecology education research, which is help to better evaluated the ability of residents, consequently, also ensuring patient safety [3, 4].

Entrustable Professional Activities (EPAs) was first proposed by Professor Olle Ten Cate in 2005 [5]. Professional behavior, in particular, refers to the “key” behavior in clinical work. EPAs evaluation is based on the clinical workplace, emphasizing the integrity of medical behavior and the observability in the work environment, which can directly reflect the clinical ability of doctors and reflect the trust of superior doctors [5–7]. Compared with the evaluation method of competency evaluation, EPAs is more close to clinical practice and can better reflect clinical competence [8–10]. Compared with competency model evaluation represented by milestone system, EPAs is easier to be understood by residents and clinical teachers, with simple evaluation criteria and more suitable for evaluation in clinical environment [6, 11]. Since 2005, various training institutions in United States, the Netherlands and Canada have conducted a large number of studies on EPA in various stages of medical education [8, 9, 12–14].

In 2020, based on foreign research background, the first Chinese EPA items for residents was proposed by 38 clinical teaching experts from 11 teaching hospitals of Peking University Health Science Center [10]. Used this establishment system, wchich including 15 EPAs, we conducted a evaluation study based on EPAs among residents. In this study, we purposed to preliminarily explore EPAs-evaluation effect, and to identify the existing problems in the clinical training of resident training in Shandong Province, so as to put forward constructive suggestions. EPAs can be used for formative evaluation and final evaluation [6, 7, 10]. Clinical teachers and teaching management departments can grasp the training situation in real time, conduct dynamic regulation and personalized training, ensure the quality of training, and finally achieve the standardized and homogenized training goals [10, 15].

Qilu hospital of Shandong University has totally 4,360 beds, more than 210,000 discharge visits, and 170,000 operations per year. In department of Gynecology and Obstetrics, there are 357 beds, including gynecologic oncology, minimally invasive gynecology, gynecologic urology, gynecologic endocrinology, perinatal medicine, family planning, reproductive medicine seven specialties. The annual outpatients of obstetrics and gynecology is more than 270,000, including a considerable number of cases are critical or difficult cases. There was a total number of more than 18,000 inpatients and 3400 deliveries in 2023.

## Methods

In our research, we took all the residents in Department of Obstetrics and Gynecology into our study. Totally, 147 residents were involved, including 100 residents who were in the rotational training, as well as 47 graduated residents who just finished standardized training. We conducted a cross-sectional study with these 147 residents, and finally 132 residents finished both self-assessment and director-assessment by internet questionnaire of Wenjuanxing, a network questionnaire website. There are 15 residents did not have both self-assessment and director-assessment because some reasons. Among these 15 residents, 7 graduated residents did not reply the self-assessment, 5 residents had only 1 piece of director-assessment and 3 residents were rotating in other departments (Ultrasonic Department and Anesthesiology Department).

### Setting

According to the national guidelines for standardized residency training, every resident of Gynecology and Obstetrics Department is supposed for different postgraduate years (PGYs), including PGY1 (who has been residency training lease than 3 months), PGY2 (who has been residency training more than 1 year but less than 15 months), PGY3 (who has been residency training more than 2 year but less than 27 months), PGY4 (who has just finished the 3-year training phase). We Create wechat groups for each PGY, then collected the self-assessment questionnaires from residents of each PGY through each wechat group. Finally 132 residents finished both self-assessment and more than 2 pieces of director-assessments. Directors in charge of Gynecologic and Obstetrics were well-trained by the national or provincial director course for Chinese standardized training residency program.

### Subjects and participants

This study enrolled 132 out of 147 residents who were trained in Gynecology and Obstetrics of a standardized residency training program and got both self-assessment and director-assessment at November 2022 apt the Qilu Hospital of Shandong University. In total, 47 directors in charge of Gynecology and Obstetrics over the same period were also recruited for our study. All the enrolled residents were categorized into PGY1 to PGY4, according to their seniority.

### Data collection

All the self-assessment and director-assessment were collected by internet questionnaire of Wenjuanxing, a network questionnaire website. The residents who had only self-assessments or director-assessment were excluded. Each residents were gathered by Wechat group when they start the by PGY were standardized residency training program in our department of Gynecology and Obstetrics. So it helped to contact graduates who finished the standardized residency training program in Gynecology and Obstetrics.

### Sample

In our research, a total of 100 residents trainees were involved, as well as 47 residents graduates. We conducted a cross-sectional study with these 147 residents, and finally 132 residents finished both self-assessment and director-assessment by internet questionnaire of Wenjuanxing, a network questionnaire website. EPAs-evaluation were referred to EPA scale of Peking Union Medical College Hospital, shown in Table 1. The scoring method is shown in Table 2.

**Table 1:** Entrustable professional activities (EPAs) categories

Number	Category
1	Admit a patient
2	Select and interpret auxiliary examinations
3	Diagnose and make the differential diagnosis
4	Make therapeutic decision
5	Compose medical documents
6	Report a case
7	Recognize and manage general clinical conditions
8	Recognize and manage emergent and critical conditions
9	Transfer and hand over a patient
10	Perform informed consent
11	Perform basic operation
12	Perform health education
13	Inform bad news
14	Perform clinical education
15	Manage public health events

**Table 2:** Levels of each entrustable professional activity (EPA)

Scale details	
1	Cannot perform certain professional activities as a resident under the direct supervision of a superior physician
2	Perform certain professional activities with a superior physician together
3	Perform certain professional activities under the supervision and guidance of a superior physician
4	Perform certain professional activities without the presence of a superior physician; when help is needed, need the presence of a superior physician to recheck all performances.
5	Perform certain professional activities without the presence of a superior physician; when help is needed, need the presence of a superior physician to recheck important performances.
6	Perform certain professional activities without the presence of the superior physician; when help is needed, need the guidance and recheck of superior physician over the phone.
7	Perform certain professional activities without the need for supervision and guidance from a superior physician.
8	Can provide supervision and guidance for others in certain professional activities.

### Ethical approvals

The study was approved by the Qilu Hospital of Shandong University Institutional Review Board.

## Statistics

Statistical software IBM SPSS (version 23.0) was used. All the questionnaires were administered using the Wenjuanxing website (https://www.wjx.cn/). Data was collected by using Excel (Microsoft, Redwood, WA, United States), and statistical analyzed by using SPSS (version 23.0). All the scores of assessments were described in Exp. and Std. Kruskal–Wallis test was used in comparisons between self-assessments and director-assessments for each EPA out of different PGYs. Mann–Whitney U test was used for each EPA assessments between every two different postgraduate years (PGYs). Analysis of PGY and identify position on the EPA scores of director assessments was analyzed by generalized estimated equation (GEE). The *p* value of 0.05 was considered statistically significant.

## Results

### The influence of PGYs and training status on EPA scores

#### Characteristics of residents and questionnaires

In this study, total of 399 questionnaires of assessment were collected, including 132 self-assessment and 267 director-assessment questionnaires. Enrolled residents were categorized into PGY1 to PGY4, according to their seniority. Number and percentage of residents, director-assessment questionnaires, self-assessment were shown in Table 3. For each residents, an average of more than 2 director-assessments were obtained to partly avoid subjective factors. We use the average score of director-assessments for statistical analysis.

**Table 3:** Characteristics of residents and questionnaires

Characteristics	PGY1	PGY2	PGY3	PGY4	Total
Number of residents, n(%)	25(18.94%)	32(24.24%)	39(29.55%)	36(27.27%)	132
Number of director-assessment questionnaires, n(%)	52(19.48%)	66(24.72%)	86(32.21%)	63(23.60%)	267
Number of self-assessment	25(18.94%)	32(24.24%)	39(29.55%)	36(27.27%)	132
Number of director-assessment for each residents (mean,SD)	2.85 ± 1.06	2.80 ± 1.23	2.60 ± 1.3	2.48 ± 1.18	
Number of different position					
Social training residents	1	1	3	0	5
Entrusted training residents	7	8	11	11	37
Professional master residents	17	17	24	21	79
Permanent staff	1	1	5	4	11

#### Higher seniority has a higher EPA score in director-assessment

**Fig. 1:**
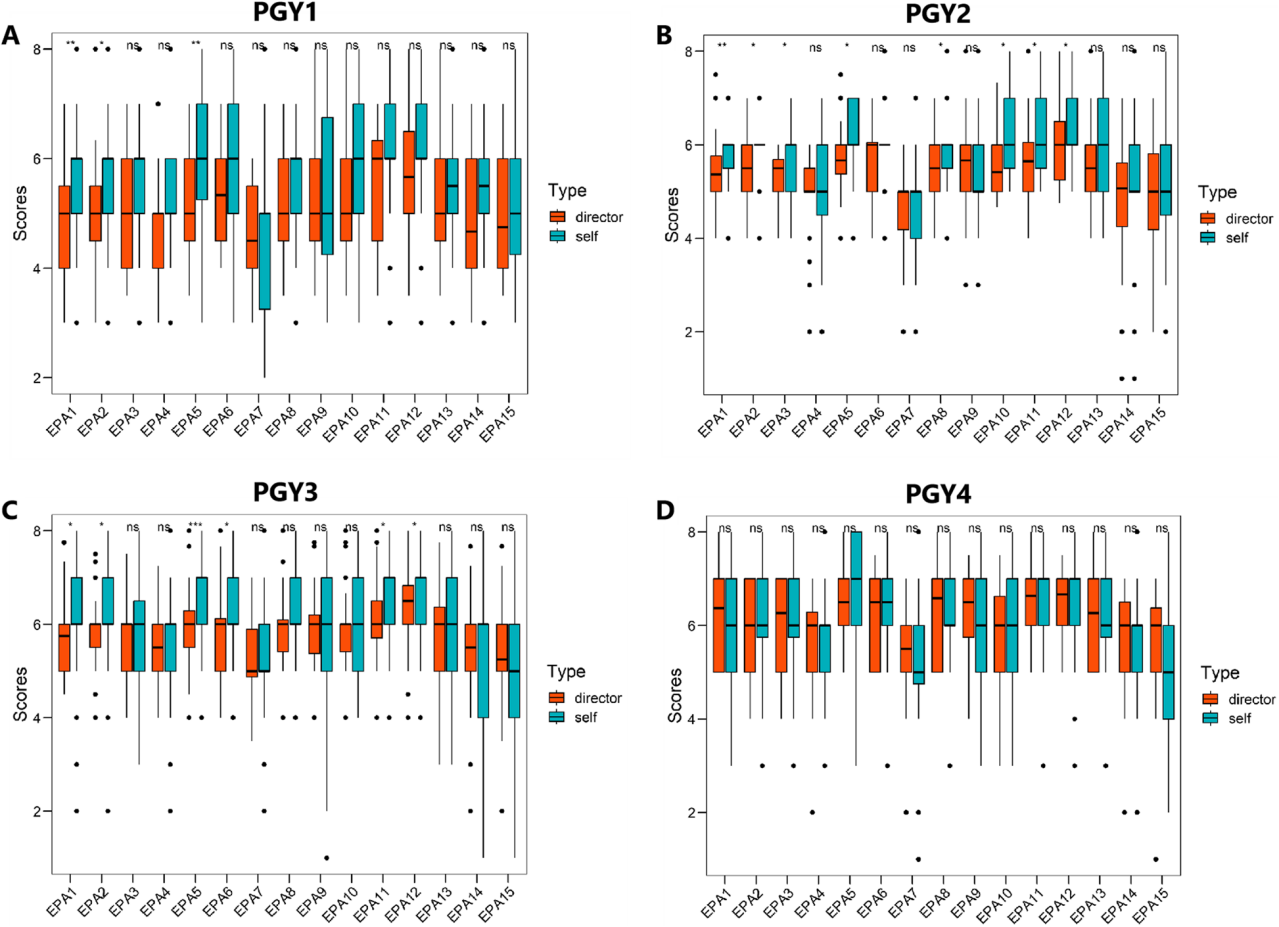
Comparison of Director-assessment in EPAs of different PGYs between self-assessment and director-assessment. **A** Comparison of Director-assessment in EPAs of PGY1 between self-assessment and director-assessment. **B** Comparison of Director-assessment in EPAs of PGY2 between self-assessment and director-assessment. **C** Comparison of Director-assessment in EPAs of PGY3 between self-assessment and director-assessment. **D** Comparison of Director-assessment in EPAs of PGY4 between self-assessment and director-assessment. * *p* < 0.05; **p < 0.01, ***p < 0.001

Date of director-assessment showed that higher seniority has a higher EPA scores (*p* < 0.05, Table 4 and Fig. 1). In detail, compared to PGY4, there were 6 EPAs of PGY1, 10 EPAs of PGY2 and 1 EPAs of PGY3 had significant difference (*p* < 0.05, shown in Table 5).

**Table 4:** Scores of director-assessment in different postgraduate years (PGYs)

EPAs	PGY1	PGY2	PGY3	PGY4	Total	Chi-square	*p* value
EPA1	4.87 ± 1.01	5.38 ± 0.76	5.76 ± 0.75	6.24 ± 0.97	5.63 ± 0.98	27.895	0.0001
EPA2	5.04 ± 1.05	5.45 ± 0.74	5.79 ± 0.76	6.06 ± 0.92	5.64 ± 0.92	20.237	0.0002
EPA3	5.00 ± 0.92	5.28 ± 0.64	5.69 ± 0.83	6.07 ± 0.94	5.56 ± 0.92	22.199	0.0001
EPA4	4.74 ± 0.93	4.90 ± 0.95	5.59 ± 0.74	5.66 ± 1.00	5.28 ± 0.98	26.066	0.0001
EPA5	5.19 ± 0.91	5.73 ± 0.71	5.95 ± 0.84	6.35 ± 0.88	5.86 ± 0.92	22.389	0.0001
EPA6	5.34 ± 1.00	5.73 ± 0.81	5.81 ± 0.87	6.25 ± 0.87	5.82 ± 0.92	13.686	0.0034
EPA7	4.56 ± 0.97	4.64 ± 0.86	5.26 ± 0.79	5.41 ± 1.03	5.01 ± 0.97	19.211	0.0002
EPA8	5.20 ± 1.06	5.47 ± 0.74	5.86 ± 0.75	6.29 ± 0.90	5.76 ± 0.94	21.220	0.0001
EPA9	5.13 ± 1.05	5.54 ± 1.03	5.83 ± 0.80	6.27 ± 0.92	5.75 ± 1.01	21.805	0.0001
EPA10	5.30 ± 1.09	5.64 ± 0.76	5.87 ± 0.86	5.96 ± 1.00	5.73 ± 0.95	10.176	0.0171
EPA11	5.54 ± 1.08	5.72 ± 0.89	6.10 ± 0.85	6.39 ± 0.83	5.98 ± 0.95	14.999	0.0018
EPA12	5.80 ± 1.29	5.95 ± 0.81	6.34 ± 0.89	6.41 ± 0.84	6.16 ± 0.97	10.682	0.0136
EPA13	5.22 ± 1.15	5.42 ± 0.88	5.75 ± 1.00	6.17 ± 0.91	5.68 ± 1.03	16.128	0.0011
EPA14	4.79 ± 1.18	4.86 ± 1.32	5.50 ± 1.06	5.74 ± 1.08	5.28 ± 1.21	17.130	0.0007
EPA15	4.96 ± 0.99	4.90 ± 1.25	5.34 ± 1.07	5.62 ± 1.18	5.24 ± 1.16	10.216	0.0168

**Table 5:** Generalized estimated equation analysis of director-assessment questionnaires

EPAs	Factor	Parameter	Exp(b)	95% confidence interval	*p* value
EPA1	PGY	PGY1	0.255	0.092 ~ 0.701	0.008
		PGY2	0.423	0.204 ~ 0.876	0.021
		PGY3	0.617	0.329 ~ 1.158	0.133
		PGY4	Refer. value		
	Position	Social training residents	0.357	0.212 ~ 0.603	0.000
		Entrusted training residents	0.555	0.283 ~ 1.087	0.086
		Professional master	0.351	0.182 ~ 0.675	0.002
		Permanent staff	Refer. value		
EPA2	PGY	PGY1	0.360	0.156 ~ 0.834	0.017
		PGY2	0.541	0.226 ~ 1.297	0.169
		PGY3	0.759	0.450 ~ 1.283	0.304
		PGY4	Refer. value		
	Position	Social training residents	0.328	0.243 ~ 0.441	0.000
		Entrusted training residents	0.500	0.321 ~ 0.777	0.002
		Professional master	0.331	0.222 ~ 0.495	0.000
		Permanent staff	Refer. value		
EPA3	PGY	PGY1	0.345	0.141 ~ 0.843	0.020
		PGY2	0.454	0.213 ~ 0.966	0.040
		PGY3	0.685	0.392 ~ 1.199	0.185
		PGY4	Refer. value		
	Position	Social training residents	0.580	0.147	0.147
		Entrusted training residents	0.732	0.303	0.303
		Professional master	0.425	0.009	0.009
		Permanent staff	Refer. value		
EPA4	PGY	PGY1	0.401	0.158 ~ 1.048	0.062
		PGY2	0.469	0.243 ~ 0 .905	0.024
		PGY3	0.939	0.569 ~ 1.550	0.805
		PGY4	Refer. value		
	Position	Social training residents	0.369	0.258 ~ 0.526	0.000
		Entrusted training residents	0.540	0.349 ~ 0.835	0.006
		Professional master	0.295	0.183 ~ 0.474	0.000
		Permanent staff	Refer. value		
EPA5	PGY	PGY1	0.313	0.137 ~ 0.716	0.006
		PGY2	0.535	0.304 ~ 0.942	0.030
		PGY3	0.666	0.419 ~ 1.058	0.085
		PGY4	Refer. value		
	Position	Social training residents	0.399	0.236 ~ 0.674	0.001
		Entrusted training residents	0.596	0.321 ~ 1.104	0.100
		Professional master	0.354	0.190 ~ 0.661	0.001
		Permanent staff	Refer. value		
EPA6	PGY	PGY1	0.401	0.163 ~ 0.982	0.046
		PGY2	0.590	0.334 ~ 1.043	0.070
		PGY3	0.642	0.417 ~ 0.988	0.044
		PGY4	Refer. value		
	Position	Social training residents	0.620	0.328 ~ 1.172	0.142
		Entrusted training residents	0.684	0.359 ~ 1.303	0.248
		Professional master	0.382	0.199 ~ 0.735	0.004
		Permanent staff	Refer. value		
EPA7	PGY	PGY1	0.424	0.161 ~ 1.120	0.083
		PGY2	0.458	0.238 ~ 0.885	0.020
		PGY3	0.857	0.542 ~ 1.357	0.511
		PGY4	Refer. value		
	Position	Social training residents	0.332	0.199 ~ 0.554	0.000
		Entrusted training residents	0.578	0.341 ~ 0.979	0.041
		Professional master	0.327	0.188 ~ 0.568	0.000
		Permanent staff	Refer. value		
EPA8	PGY	PGY1	0.335	0.113 ~ 0.988	0.047
		PGY2	0.439	0.278 ~ 0.692	0.000
		PGY3	0.649	0.392 ~ 1.074	0.092
		PGY4	Refer. value		
	Position	Social training residents	0.329	0.182 ~ 0.595	0.000
		Entrusted training residents	0.476	0.303 ~ 0.746	0.001
		Professional master	0.291	0.174 ~ 0.487	0.000
		Permanent staff	Refer. value		
EPA9	PGY	PGY1	0.320	0.092 ~ 1.108	0.083
		PGY2	0.482	0.219 ~ 1.061	0.020
		PGY3	0.645	0.369 ~ 1.127	0.511
		PGY4	Refer. value		
	Position	Social training residents	0.712	0.177 ~ 0.395	0.437
		Entrusted training residents	0.429	0.302 ~ 0 .608	0.000
		Professional master	0.264	0.303 ~ 1.675	0.000
		Permanent staff	Refer. value		
EPA10	PGY	PGY1	0.320	0.188 ~ 1.395	0.191
		PGY2	0.723	0.422 ~ 1.240	0.239
		PGY3	0.909	0.554 ~ 1.491	0.706
		PGY4	Refer. value		
	Position	Social training residents	0.611	0.322 ~ 1.159	0.131
		Entrusted training residents	0.451	0.290 ~ 0.700	0.000
		Professional master	0.293	0.198 ~ 0.435	0.000
		Permanent staff	Refer. value		
EPA11	PGY	PGY1	0.425	0.149 ~ 1.215	0.110
		PGY2	0.509	0.277 ~ 0.935	0.030
		PGY3	0.748	0.445 ~ 1.259	0.275
		PGY4	Refer. value		
	Position	Social training residents	0.538	0.194 ~ 0.452	0.151
		Entrusted training residents	0.485	0.303 ~ 0.775	0.003
		Professional master	0.296	0.230 ~ 1.255	0.000
		Permanent staff	Refer. value		
EPA12	PGY	PGY1	0.545	0.185 ~ 1.609	0.272
		PGY2	0.632	0.405 ~ 0.984	0.042
		PGY3	0.938	0.635 ~ 1.387	0.749
		PGY4	Refer. value		
	Position	Social training residents	0.711	0.334 ~ 1.516	0.378
		Entrusted training residents	0.484	0.280 ~ 0.837	0.009
		Professional master	0.292	0.183 ~ 0.469	0.000
		Permanent staff	Refer. value		
EPA13	PGY	PGY1	0.385	0.133 ~ 1.120	0.080
		PGY2	0.472	0.219 ~ 1.017	0.055
		PGY3	0.657	0.416 ~ 1.039	0.073
		PGY4	Refer. value		
	Position	Social training residents	1.00	0.234 ~ 4.315	0.995
		Entrusted training residents	0.616	0 .331 ~ 1.146	0.126
		Professional master	0.400	0.218 ~ 0.735	0.003
		Permanent staff	Refer. value		
EPA14	PGY	PGY1	0.385	0.491 ~ 1.262	0.100
		PGY2	0.412	0.235 ~ 0.721	0.002
		PGY3	0.787	0.124 ~ 1.201	0.320
		PGY4	Refer. value		
	Position	Social training residents	0.345	0.103 ~ 1.152	0.084
		Entrusted training residents	0.533	0.258 ~ 1.104	0.090
		Professional master	0.298	0 .169 ~ 0.526	0.000
		Permanent staff	Refer. value		
EPA15	PGY	PGY1	0.517	0.169 ~ 1.583	0.248
		PGY2	0.486	0.231 ~ 1.023	0.057
		PGY3	0.752	0.414 ~ 1.365	0.248
		PGY4	Refer. value		
	Position	Social training residents	0.676	0.231 ~ 1.979	0.475
		Entrusted training residents	0.569	0.275 ~ 1.178	0.129
		Professional master	0.302	0.157 ~ 0.581	0.000
		Permanent staff	Refer. value		

There was a trend of growth in self-assessment of residents’ training, but no significance was found in statistic of our study (Table 6).

**Table 6:** Scores of self-assessment in different postgraduate years (PGYs)

EPAs	PGY1	PGY2	PGY3	PGY4	Total	Chi-square	*p* value
EPA1	5.69 ± 1.09	5.89 ± 0.75	6.16 ± 1.34	6.22 ± 1.24	6.03 ± 1.07	4.854	0.1828
EPA2	5.65 ± 1.02	5.78 ± 0.70	6.07 ± 1.22	5.94 ± 1.19	5.89 ± 1.09	4.251	0.2356
EPA3	5.53 ± 1.03	5.70 ± 0.67	5.86 ± 1.13	6.08 ± 1.18	5.82 ± 1.05	6.111	0.1063
EPA4	5.04 ± 1.19	5.11 ± 1.31	5.46 ± 1.14	5.58 ± 1.18	5.34 ± 1.19	5.092	0.1652
EPA5	5.96 ± 1.15	6.15 ± 0.77	6.72 ± 0.98	6.50 ± 1.36	6.39 ± 1.12	9.298	0.0256
EPA6	5.69 ± 1.29	5.96 ± 0.89	6.33 ± 1.04	6.36 ± 1.20	6.13 ± 1.13	6.883	0.0757
EPA7	4.69 ± 1.52	4.85 ± 1.06	5.04 ± 1.27	5.02 ± 1.52	4.93 ± 1.35	2.145	0.5428
EPA8	5.62 ± 1.10	5.96 ± 0.90	6.23 ± 0.90	6.36 ± 1.05	6.09 ± 1.00	9.363	0.0248
EPA9	5.46 ± 1.63	5.56 ± 1.31	5.93 ± 1.55	6.03 ± 1.34	5.79 ± 1.47	4.022	02591
EPA10	5.77 ± 1.14	6.22 ± 0.93	6.13 ± 0.99	5.97 ± 1.53	6.04 ± 1.18	1.992	0.5741
EPA11	6.08 ± 1.16	6.33 ± 1.07	6.51 ± 1.01	6.51 ± 1.11	6.39 ± 1.08	3.079	0.3797
EPA12	6.23 ± 1.18	6.48 ± 1.01	6.72 ± 0.88	6.67 ± 1.17	6.56 ± 1.06	4.061	0.2550
EPA13	5.42 ± 150	5.96 ± 1.16	6.14 ± 1.21	6.19 ± 1.21	5.98 ± 1.28	5.827	0.1203
EPA14	5.27 ± 1.31	5.41 ± 1.55	5.21 ± 1.66	5.64 ± 1.17	5.38 ± 1.44	0.982	0.8055
EPA15	5.27 ± 1.51	5.15 ± 1.46	5.02 ± 1.65	5.17 ± 1.44	5.13 ± 1.52	0.244	0.9702

#### Different EPAs seniority had different EPA score

The scores for different EPA projects were different. Among these 15 EPAs, residents got relatively low scores in EPA4 (Make therapeutic decision, total score of 5.28 ± 0.98), EPA7 (Recognize and manage general clinical conditions, total score of 5.01 ± 0.97), EPA14 (Perform clinical education, total score of 5.28 ± 1.21) and EPA15 (Manage public health events, total score of 5.24 ± 1.16). It was shown in Table 4.

#### Significant difference were found between different position

**Fig. 2:**
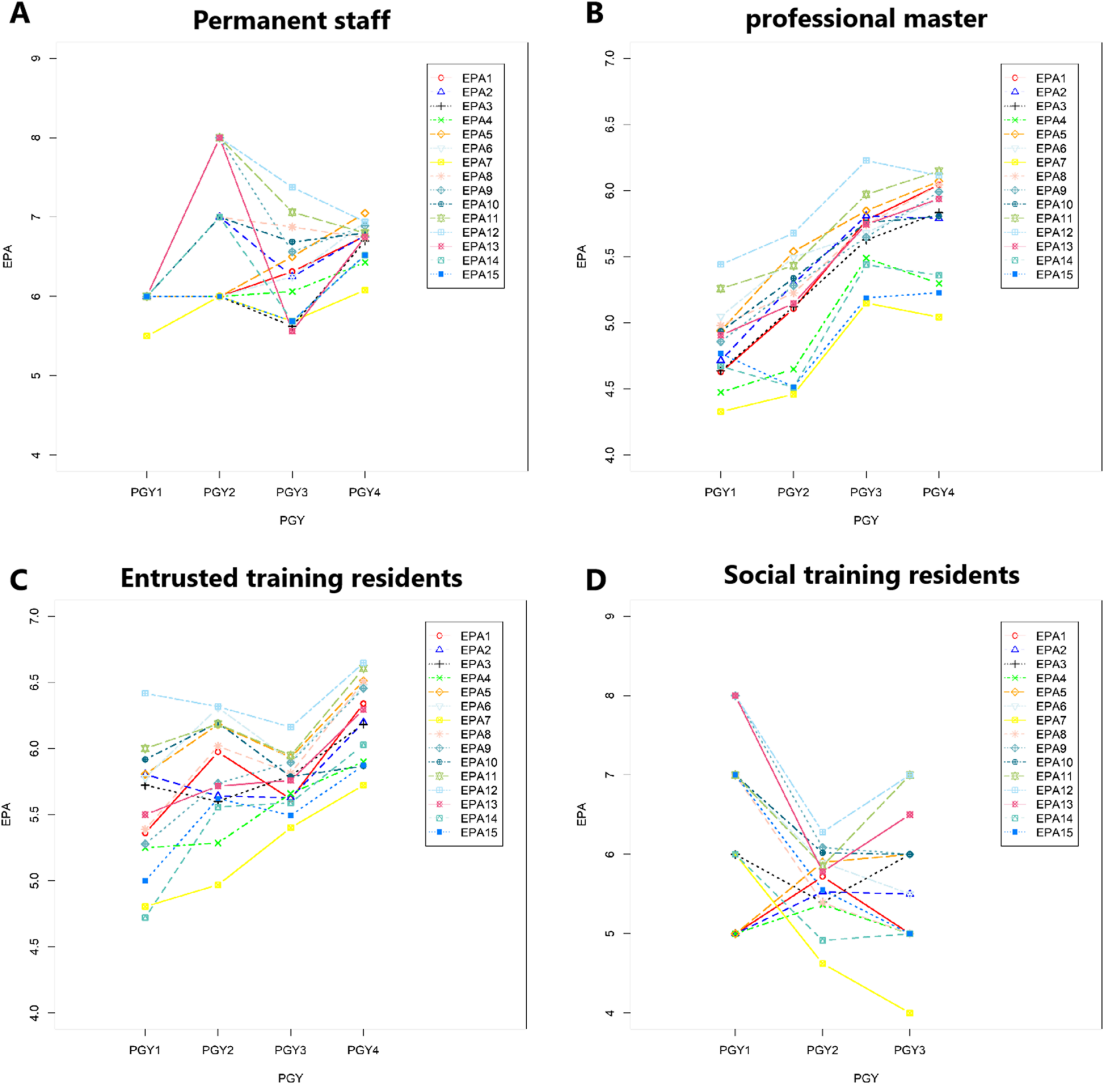
Director-assessment of different position. **A** Director-assessment of EPAs in permanent staff of different PGYs. **B** Director-assessment of EPAs in professional master of different PGYs. **C** Director-assessment of EPAs in entrusted training residents of different PGYs. **D** Director-assessment of EPAs in social training residents of different PGYs

Among the four different position of residents (social training residents, entrusted training residents, professional master and permanent staff), permanent staff has the highest EPA scores in this EPAs-evaluation study. Compared to permanent staff, 6 EPAs showed significance in social training residents, 8 EPAs showed significant difference in entrusted training residents (*p* < 0.05). Professional master showed the least desirable EPA scores in all of these 15 EPAs (shown in Table 6 and Fig. 2).

#### Significant difference was found in PGYs between self-assessment and director-assessment.

**Fig. 3:**
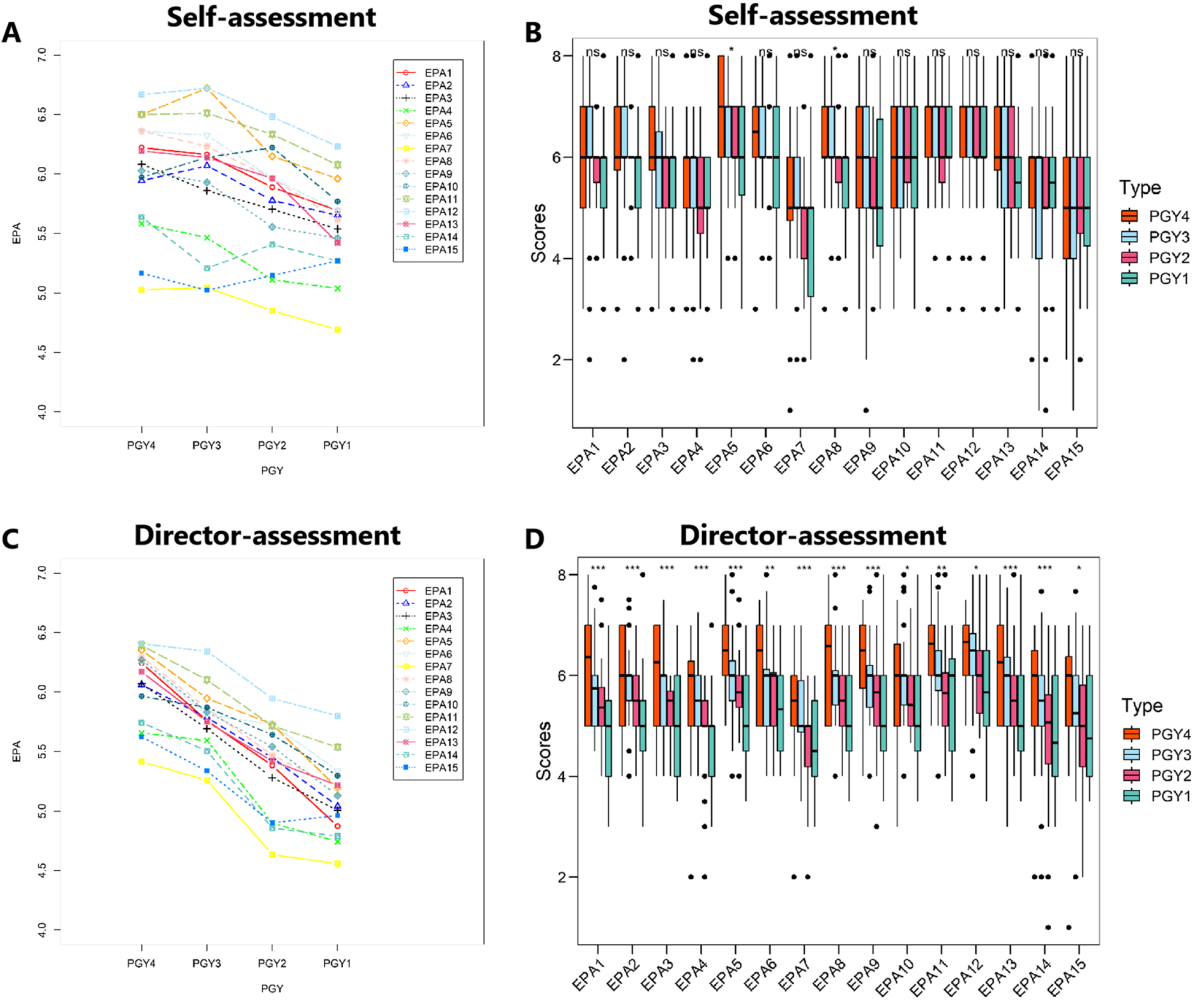
Director-assessment of different seniority (different postgraduate years). **A** Self-assessment of different PGYs. **B** Statistic of different PGYs in self-assessment productive history of patients. It show the gestation, abortion, and partus maturus. **C** Director-assessment of different PGYs. **D** Statistic of different PGYs in director-assessment. * *p* < 0.05; **p < 0.01, ***p < 0.001

In this study, we found significant difference between self-assessment and director-assessment in PGY1, PGY2 and PGY3 (*p* < 0.05). In detail, there were 8 EPAs found had significant higher scores of self-assessment than that of director-assessment in PGY2, meanwhile, 6 EPAs in PGY3, and EPAs in PGY1 (Fig. 3). However, for PGY4 residents, self-assessment and director-assessment results were relatively consistent. Self-assessment sores of PGY1 residents were significant higher in 3 EPAs than director-assessment scores, what is more, no significance was found in PGY4 residents (Fig. 3).

#### Different EPA evaluation between self- and director- assessment

Compared with director-assessment, significant difference was found in self-assessment on admit a patient, select and interpret auxiliary examinations, compose medical documents, perform basic operation and perform health education (shown in Fig. 3).

## Discussion

In China, the standardized training of resident doctors (including the training of clinical medical degree) is an important part of post-graduation medical education [10]. It plays a role in medical school education and resident education, which determines the career development direction of young doctors. Evaluation of residents’ competency has always been the focus and difficulty of standardized training. Kinds of evaluation, including mini-clinical-evaluation exercises (Mini-CEX) and direct observation of procedural skills (DOPS), had been performed for decades [16, 17], however educational impact varies greatly and could be influenced by a few factors [18, 19]. Entrustable professional activities (EPAs) is a new competency model which includes the clinical key familiar behavior for teachers and residents to evaluate the comprehensive ability of residents in daily work. It has been recognized that EPAs can better reflect the abilities of residents [14, 20–22].

In this study, we used Chinese EPA scale, which has been proved its validity in Pediatric [10, 15]. We conduct a cross-sectional study towards residents and instructors in Gynecology and Obstetric. Residents were thought to enhance their clinical professional skills as the training time increased [23]. Our results indeed showed that the clinical competence of residents was gradually enhanced year by year according to whether self-assessment or director-assessment. For the trend of competence growth, an obviously growing peak was found at PGY2, which mean students of PGY2 made great progress during their residential training. Compared to director-assessment, residents of PGY1, PGY2 and PGY3 had more or less over-confidence. But self-assessment of PGY4 residents were almost consistent to director-assessment. This result indicated that residents may make mistakes in performing clinical professional behavior based on overconfidence, which should be paid attention during clinical work.

As director-assessment was much more accurate, we did exact statistics on director-assessment. Among different PGYs, standard deviation of PGY1 was more than that of other PGYs on most of EPAs, except EPA3 (Diagnose and make the differential diagnosis), EPA4 (Make therapeutic decision) and EPA15 (Manage public health events). It indicated that graduated medical students had different ability in those EPAs, however relatively consistent ability in these 3 EPAs above. The statistic date of director-assessment show deficiencies in EPA4 (Make therapeutic decision), EPA7 (Recognize and manage general clinical conditions), EPA11 (Perform clinical education) and EPA12 (Manage public health events), which need to be improved in future resident training.

Among different position of residents, permanent staff has the highest EPA scores in this EPAs-evaluation study. The reason might be that permanent staff had higher education degree, and more stronger sense of responsibility and ownership. What’s more, permanent staff might also have better initiative and exercise opportunity. However, residents of professional master showed the least desirable EPA scores in all of these 15 EPAs, which was contribute to deficiencies in clinical practice. Social training residents and entrusted training residents usually had a period of work experience, which result in a better competency basis. But it finally show similar ability score in residents of social training, entrusted training and professional master, except permanent staff. Nevertheless, of the residents group, the social training and the relative number of staff in our hospital were relatively small, which may have statistical bias.

There were also several limitations in this current study. First, as this is a cross-sectional study, more residents could be involved in future study. Second, the study was a cross-sectional study that residents of different PGYs may have individual differences, which may affects the statistical results. A longitudinal study To survey skill assessment of the same residents during the training time may be better for trend research. However, in our study, however growing trend of EPAs still could be concluded according to EPA scores of differen PGYs. Third, there was partly difference between different directors, which we did detailed pre-assessment training to avoid this. Last, only single centre of Qilu hospital was involved. Multiple centre will be joined in this evaluation research.

There were also several limitations in this current study. First, the PGY1 residents who were involved in this study had different training rotate in the first 3 months, which may lead to a certain difference in both self-assessment and director-assessment. But it will be gradually reduced in subsequent PGYs’ assessments. Second, the directors of each resident are not the same. Although we reduce this bias by including two or more directors for assessment, there was still a certain subjective bias. A more comprehensive and detailed process evaluation system will be carried out in future studies.

## Conclusion

In conclusion, for evaluation of residents’ competency, we put forward a novel model of EPA assessment to evaluate the quality of residential training teaching and improve the standardization training process of Chinese resident doctors. Our model of EPA assessment show a good evaluation efficiency, and more easier to handle, indicating a broad prospect.

## Supplementary Information

Supplementary Material 1.

Supplementary Material 2.

## Data Availability

All data generated or analyzed during this study are included in this published article and its supplementary information files.

## Abbreviations

DOPS: Direct observation of procedural skills
EPA: Entrustable professional activity
Mini-CEX: Mini-clinical-evaluation exercises
OB &GYN: Obstetrics and Gynecology
PGY: Postgraduate year
